# Prevalence of Bullying, Discrimination, and Harassment Among Women in Cardiology: A Snapshot Australasian Survey

**DOI:** 10.1016/j.jacadv.2026.102866

**Published:** 2026-07-22

**Authors:** Jaya Chandrasekhar, Swati Mukherjee

**Affiliations:** a Department of Cardiology, Box Hill Hospital, Eastern Health, Melbourne, Victoria, Australia; b Eastern Health Clinical School, Monash University, Melbourne, Victoria, Australia; c Department of Cardiology, Cabrini Health, Melbourne, Victoria, Australia; d The School of Translational Medicine, Monash University, Melbourne, Victoria, Australia

**Keywords:** bias, bullying, discrimination, gender disparities, sexual harassment

## Abstract

**Background:**

Bullying, discrimination, and harassment (BDH) are prevalent in medicine and are more commonly experienced by women.

**Objectives:**

The Australia New Zealand Women in Cardiology (WIC) working group sought to assess the current prevalence of BDH among females in cardiology through a snapshot survey.

**Methods:**

The survey was developed by 2 women cardiologists and distributed to members of the WIC email list (180 members). The survey was also rolled out at a simultaneously held WIC meeting in Sydney and Melbourne, Australia, in April 2024. The data were received anonymously and summary data tabulated and presented.

**Results:**

The survey was completed by 105 individuals (58.33% completion rate). Approximately 89.52% reported experiencing at least 1 negative behavior at any stage in their career, and 59.1% reported at least 1 negative behavior within the last 12 months. Majority (75.2%) of these behaviors were displayed by cardiology consultants. Of the respondents, 29.5% did not know how to react to the behavior and 17.1% reported the behavior higher up but no action was taken. Nearly half (47.6%) of the respondents were worried about the effect of reporting on day-to-day work or training. About 39.1% felt the hospital executive would be most effective in minimizing BDH.

**Conclusions:**

BDH was noted to be highly prevalent from this snapshot survey of women in cardiology. Urgent measures are required to mitigate and abolish these negative behaviors, a process that requires significant changes from all stakeholders involved in cardiology workplaces.

Bullying, discrimination, and harassment (BDH) are pervasive issues in professional workplaces including medicine and cardiology. Health care professionals can face significant challenges related to negative workplace culture. These adverse behaviors can take the form of gender-based discrimination, racial and ethnic bias, or inappropriate conduct impacting the psychological, physical, and emotional well-being of those involved as well as affecting their capacity to work effectively.^1^ Understanding the dynamics and knock-on effect of BDH in cardiology is critical to creating safer, more inclusive work environments, and ensuring optimal patient care.

Following a sentinel event involving the suspension of an internationally renowned cardiologist in relation to sexual harassment allegations reported in early 2024, we developed this pilot study.^2^ The aim of the current snapshot survey was to assess the current prevalence of BDH among women in cardiology in Australasia, with a view to subsequently working with stakeholders for corrective measures. To our knowledge, no BDH survey has been conducted to date in this cohort of the region.

## Methods

Two members of the Australia New Zealand Women in Cardiology (WIC) working group (authors J.C. and S.M.) independently designed a pilot survey using SurveyMonkey (SurveyMonkey Inc) to capture snapshot information regarding BDH among women in the cardiology community in Australia and New Zealand. The survey was distributed via email to 180 women on the WIC mailing list and made available through a Quick Response code at the WIC dinner, held concurrently in Sydney and Melbourne in April 2024. The survey was open for a period of 2 months and responses were received anonymously. Ethics approval for this study was retrospectively obtained from the Eastern Health Ethics Committee.

The survey had 4 parts (Table 1):

1. Demographic questions (4 questions related to age, gender, location, and membership status with Cardiac Society of Australia and New Zealand [CSANZ])
2. Experience of BDH (4 questions related to experience ever in the workplace, experience within the last 12 months, perpetrators of the behavior, and responses to the behavior)
3. Questions related to drivers/barriers to cardiology practice and capacity to minimize bias (4 questions)
4. Questions related to periodic surveys (2 questions)

**Table 1:** Survey Questions and Responses

Question	Answer Choices	Response Rate
1. My age	Under 30	3.81%
	31-40	36.19%
	41-50	32.38%
	51-60	20.00%
	61-70	3.81%
	Above 70	3.81%
2. I describe my gender as	Male	0.95%
	Female	99.05%
	In another way	0.00%
3. My location	Australia	98.10%
	New Zealand	1.90%
4. My status with CSANZ (please tick 1)	Fellow <10 years	45.71%
	Fellow >10 years	32.38%
	Cardiology trainee	2.86%
	Specialist international medical graduate	0.00%
	Nurse	4.76%
	Other	11.43%
	Prefer not to answer	2.86%
5. Thinking about your workplace, have you personally experienced or witnessed in your department any of the following behaviors ever in your cardiology career? (Please tick all that apply)	Aggression or physical abuse	20.95%
	Being undermined/humiliating comments made about me or toward me or a colleague when alone	60.00%
	Humiliating comments made about me or toward me or a colleague in front of others	53.33%
	Inappropriate criticisms/accusations/belittling behavior	62.86%
	Yelling or shouting	41.90%
	Unwelcome advances of a sexual nature (breach of personal space)	31.43%
	Being denied operating lists or procedures	12.38%
	Being denied training opportunities	23.81%
	Being excluded from meetings related to my role	16.19%
	Being assigned meaningless tasks	19.05%
	Being excluded from social events where other colleagues have been invited	29.52%
	Being denied a promotion	15.24%
	Being denied an interview opportunity for a leadership role	8.57%
	Receiving favorable/unfavorable treatment because of gender/race	28.57%
	Comments about my culture or race that made me feel uncomfortable	14.29%
	Other unwelcome behavior	17.14%
	Opinions and views ignored	39.05%
	Pressured into not claiming something to which you were entitled (e.g. overtime payment, leave, reimbursement, increased paygrade, faculty position)	47.62%
	Suggested to quit job/training program	8.57%
	None of the above	10.48%
6. Thinking about your workplace, have you personally experienced or witnessed in your department any of the following behaviors in the last 12 months? (Please tick all that apply)	Aggression or physical abuse	3.81%
	Being undermined/humiliating comments made about me or toward me or a colleague when alone	29.52%
	Humiliating comments made about me or toward me or a colleague in front of others	24.76%
	Inappropriate criticisms/accusations/belittling behavior	24.76%
	Yelling or shouting	13.33%
	Unwelcome advances of a sexual nature (breach of personal space)	5.71%
	Being denied operating lists or procedures	7.62%
	Being denied training opportunities	7.62%
	Being excluded from meetings related to my role	8.57%
	Being assigned meaningless tasks	11.43%
	Being excluded from social events where other colleagues have been invited	6.67%
	Being denied a promotion	8.57%
	Being denied an interview opportunity for a leadership role	5.71%
	Receiving favorable/unfavorable treatment because of gender/race	12.38%
	Comments about my culture or race that made me feel uncomfortable	5.71%
	Other unwelcome behavior	10.48%
	Opinions and views ignored	19.05%
	Suggested to quit job/training program	1.90%
	pressured into not claiming something to which you were entitled (e.g. overtime payment, leave, reimbursement, increased paygrade, faculty position)	14.29%
	None of the above	40.95%
7. Who displayed this behavior?	Cardiology consultant	75.24%
	Other medical consultant	13.33%
	Cardiothoracic surgical consultant	18.10%
	Nurse	12.38%
	Allied health professional	1.90%
	Medical/hospital administrator	10.48%
	Other	12.38%
	Not applicable	15.24%
8. Did any of the following influence your decision on how to respond to this behavior? (Please tick all that apply)	Not applicable	20.00%
	Did not recognize the behavior as inappropriate at the time, and accepted it as part of the norm	24.76%
	Was unsure but didn’t have anyone to talk to and debrief on the episode	23.81%
	Did not react to the behavior as I did not know how to	29.52%
	Had never heard of anyone else complaining about similar occurrences	15.24%
	Worried about the effect on my day-to-day work or training	47.62%
	Worried about the effect on my references and future career options	41.90%
	Worried about the effect on the victim's future career options (if witnessing the adverse behavior)	10.48%
	Fear of making the situation worse	42.86%
	Fear of being stigmatized	33.33%
	Reported to relevant authority but no action was taken	17.14%
	Did not want to get involved	5.71%
9. Who in your opinion makes key decisions (including but not limited to successful training completion, career options/progression) regarding professional requirements of cardiology trainees/fellows/consultants?	Hospital executive	10.48%
	Member of CSANZ (representing various institutions e.g. cardiology head of the department, cardiology advanced training state program director)	60.95%
	RACP	26.67%
	AHPRA	1.90%
10. Who in your opinion is responsible for the assessment and accreditation of an institution for providing cardiology training?	Hospital executive	4.76%
	CSANZ	28.57%
	RACP	60.00%
	AHPRA	6.67%
11. When a cardiology trainee has to move institutions especially at junior career levels what or who in your opinion limits the options	Difficulty finding another CSANZ accredited position	48.57%
	Completing annual training requirements set by CSANZ	7.62%
	RACP	6.67%
	AHPRA	0.95%
	Institution	36.19%
12. In your opinion which entity from the ones listed below holds the most capacity and thereby could be most effective in minimizing bias, discrimination, bullying, and harassment in cardiology (select a single response)	Hospital executive	39.05%
	CSANZ	33.33%
	RACP	16.19%
	AHPRA	5.71%
	None of the above	5.71%
13. Should CSANZ design and administer a work place culture survey for cardiologists and cardiology trainees, in order to obtain better data on the types of issues and their prevalence to provide better scientific oversight of the issues and of the success of any programs to address them.	Yes	90.48%
	No	3.81%
	Prefer not to answer	5.71%
14. Are you satisfied with the work being done by CSANZ currently to minimize bias, discrimination, bullying and harassment in cardiology?	Yes	27.62%
	No	72.38%
15. Any additional comments (Free text)	Shown in Figure 4	

AHPRA = Australian Health Practitioner Regulation Agency; CSANZ = Cardiac Society of Australia and New Zealand; RACP = Royal Australasian College of Physicians.

In Australia, annual medical registration is maintained by the Australian Health Practitioner Regulation Agency (AHPRA), and in New Zealand, the Medical Council of New Zealand is responsible for overseeing this. A compulsory annual fellowship fee is paid to the Royal Australasian College of Physicians (RACP) for the right to use the FRACP postnominal, and for support with continuing professional development. The CSANZ is a nongovernment, private professional body for cardiologists and individuals working in the area of cardiology. Membership or fellowship with CSANZ is optional. CSANZ has affiliations with other international counterpart organizations and focuses on professional development, educational resources, and advocacy in cardiology.

Survey responses were analyzed to provide summary statistics. Comparative analyses were performed for differences in overall BDH experiences among CSANZ fellows <10 vs >10 years. We explored for associations between awareness of BDH or having someone to talk to about the experience and reporting to a relevant authority. We examined effect sizes for reporting vs not reporting BDH based on type of BDH ever experienced. Categorical variables were compared using chi-square or Fisher exact methods. All analyses were conducted using IBM SPSS Statistics (version 29.0), and *P* values <0.05 were considered significant. We also performed qualitative analysis of free text survey comments and grouped the comments into relevant themes.

## Results

**Figure 1:**
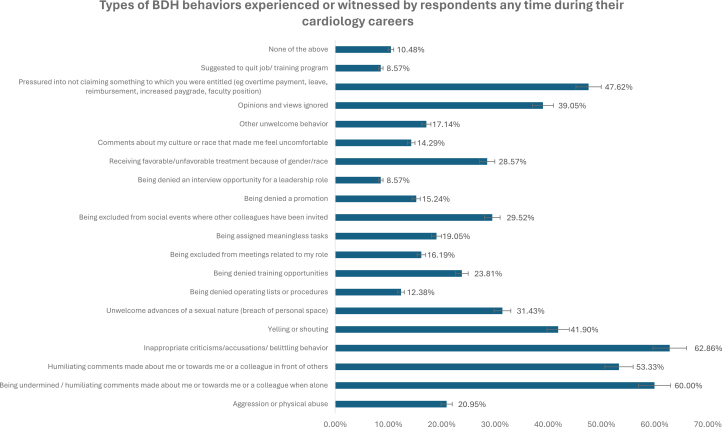
Distribution of the Types of BDH Behaviors Experienced or Witnessed by Respondents Anytime During Their Cardiology Careers BDH = bullying, discrimination, and harassment.

A total of 105 responses were received accounting for approximately 58.33% of mailing list members. Results are shown in Table 1, Figure 1 and Supplemental Tables 1 and 2.

Majority of the respondents were between 31 and 50 years of age and 99% were women. Only 2 out of 105 respondents were located in New Zealand. 45.71% had been CSANZ fellows for under a decade and 32.38% had been fellows for more than a decade.

Approximately 89.52% reported experiencing at least 1 negative behavior at any stage in their career, and 59.1% reported experiencing at least 1 negative behavior within the last 12 months. The prevalence estimates for responses to these 2 key questions in the survey are shown in Supplemental Tables 1 and 2.

Figure 1 shows the types of BDH behaviors experienced or witnessed by respondents anytime over the course of their cardiology careers. Majority (75.2%) of these behaviors were reported to be displayed by cardiology consultants followed by cardiac surgery consultants (18.1%), other medical consultants, hospital administrators, nursing, allied health staff, or others.

**Figure 2:**
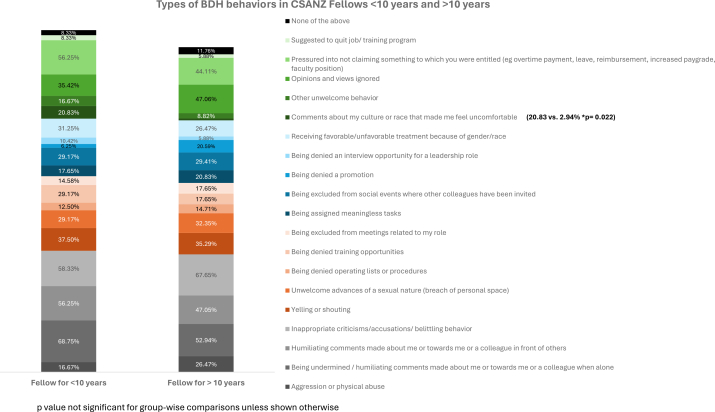
Differences in BDH Experiences Between CSANZ Fellows <10 Years and >10 Years CSANZ = Cardiac Society of Australia and New Zealand; other abbreviation as in Figure 1.

Among 82 CSANZ fellows, we compared differences in overall BDH experiences by fellow status <10 or >10 years. Non-CSANZ fellows were excluded. Results are shown in Supplemental Table 3 and Figure 2. 20.83% fellows under 10 years reported comments about culture or race that made them feel uncomfortable compared to 2.94% in fellows over 10 years (*P* = 0.022). There were no significant differences between the groups for other BDH categories ever experienced.

**Figure 3:**
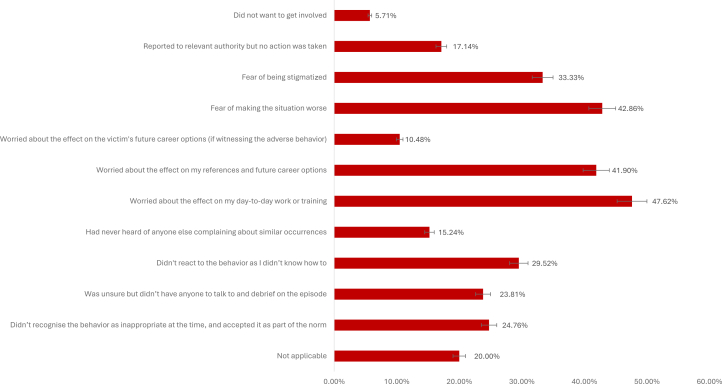
Factors Influencing Decisions on How to Respond to BDH Behavior Abbreviation as in Figure 1.

Figure 3 highlights the factors influencing respondent decisions on how to respond to BDH behavior. Approximately 24.8% respondents accepted the negative behaviors they experienced as part of the norm, 23.8% did not have anyone to talk to and debrief on the episode, 29.5% did not know how to react to the behavior, and 17.1% reported the behavior higher up but no action was taken. Nearly half (47.6%) of the respondents were worried about the effect of reporting on day-to-day work or training, 41.9% worried about the effect on references and future career options, 42.86% reported fear of making the situation worse and 33.33% reported fear of being stigmatized.

We explored for associations between awareness of how to respond to BDH or having someone to talk to and reporting without any action being taken (n = 21 cases who selected “not applicable” for the question on responding to BDH were excluded).

A total of 5/25 (20%) respondents were unsure or did not have anyone to talk to, but reported to a relevant authority, whereas 20/25, 80% of this group, were unsure and did not end up reporting the issue. In contrast, 13/59 (22.03%) respondents were aware or had someone to talk to and reported to a relevant authority. There were no significant differences in reporting rates among those who had someone to talk to about BDH compared to their counterparts (*P* = 1.00).

We also examined for associations between awareness of how to react to BDH behavior and reporting to a relevant authority. 5/31 (16.13%) respondents did not know how to react to the behavior but reported to a relevant authority with no action being taken. Conversely, 13/53 (24.53%) respondents were aware of how to react and reported to a relevant authority. There was no difference between the groups (*P* = 0.421).

The odds of reporting were significant in the case of the following types of BDH experiences as shown in Table 2: 1) experiencing aggression or physical abuse; 2) being undermined/humiliating comments made about me or towards me or a colleague when alone, or in front of others; 3) unwelcome advances of a sexual nature (breach of personal space); 4) being denied an interview opportunity for a leadership role; 5) receiving favorable/unfavorable treatment because of gender/race; 6) comments about my culture or race that made me feel uncomfortable; 7) other unwelcome behavior; and 8) opinions and views ignored.

**Table 2:** Types of BDH Experiences and Odds of Reporting to a Relevant Authority With No Action Being Taken

Thinking About Your Workplace, Have You Personally Experienced or Witnessed in Your Department Any of the Following Behaviors Ever in Your Cardiology Career? (Please Tick All That Apply)	OR for Reporting vs Not Reporting to Relevant Authority and No Action Being taken	95% CI Lower Limit	95% CI Upper Limit
Aggression or physical abuse	**3.055**	**1.018**	**9.166**
Being undermined/humiliating comments made about me or toward me or a colleague when alone	**6.809**	**1.475**	**31.419**
Humiliating comments made about me or toward me or a colleague in front of others	**9.400**	**2.037**	**43.378**
Inappropriate criticisms/accusations/belittling behavior	2.356	0.716	7.751
Yelling or shouting	2.571	0.907	7.289
Unwelcome advances of a sexual nature (breach of personal space)	**3.478**	**1.224**	**9.887**
Being denied operating lists or procedures	2.476	0.669	9.159
Being denied training opportunities	0.898	0.266	3.026
Being excluded from meetings related to my role	2.404	0.725	7.965
Being assigned meaningless tasks	1.846	0.572	5.959
Being excluded from social events where other colleagues have been invited	1.670	0.580	4.811
Being denied a promotion	2.657	0.793	8.909
Being denied an interview opportunity for a leadership role	**4.686**	**1.119**	**19.614**
Receiving favorable/unfavorable treatment because of gender/race	**4.188**	**1.458**	**12.030**
Comments about my culture or race that made me feel uncomfortable	**4.333**	**1.307**	**14.366**
Other unwelcome behavior	**4.397**	**1.407**	**13.736**
Opinions and views ignored	**2.986**	**1.049**	**8.495**
Pressured into not claiming something to which you were entitled (e.g. overtime payment, leave, reimbursement, increased paygrade, faculty position)	1.934	0.685	5.459
Suggested to quit job/training program	0.581	0.068	4.956
None of the above	0.809	0.733	0.892

Numbers in bold indicate values of statistical significance.

BDH = bullying, discrimination, and harassment.

About 39.1% felt the hospital executive would be most effective in minimizing BDH followed by CSANZ and the RACP. 72.4% were not satisfied with the work being done by CSANZ currently to minimize BDH in cardiology and 90.48% felt CSANZ should design and regularly administer a workplace culture survey to provide better scientific oversight of the issues and any programs to address them.

**Figure 4:**
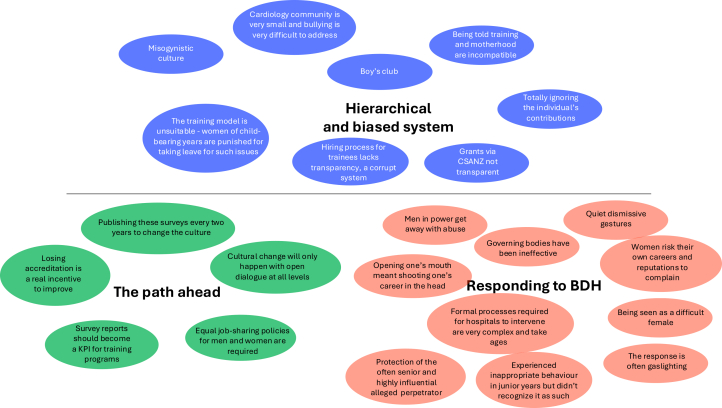
Visual Map with Thematic Representation of Free-Text Survey Comments KPI = Key Performance Indicator. Abbreviations as in Figure 1 and 3.

Figure 4 shows a visual map of free text comments from the respondents. We performed qualitative coding and presented the codes into the following themes: hierarchical and biased system (7 comments), responding to BDH (14 comments) and the path for change (6 comments).

## Discussion

Our survey examined the prevalence of BDH, including experiences across the workplace within the past 12 months, the identified perpetrators of such behaviors, and the responses to them. It also explored the drivers and barriers to a fulfilling cardiology practice for women, as well as strategies to minimize bias. Uniquely, this paper addresses the issue of discrimination by examining how cardiology colleagues perceive bias, making the findings relevant to those at all stages of their career in cardiology.

Key findings of this pilot WIC survey are as follows:

1. High prevalence of BDH: Nearly 90% of respondents reported experiencing BDH at some point in their career, with close to 60% reporting such experiences within the 12 months preceding the survey.
2. Limited awareness of reporting pathways: Approximately 1 in 4 respondents (23.8%) were either unaware of a reporting pathway in their institution or felt they had no one to speak to regarding these behaviors.
3. Concerns about professional impact: Close to half of the respondents expressed worry about the effect of reporting BDH on their day-to-day work or future career prospects.
4. Lack of corrective action: Nearly 1 in 5 respondents (17.1%) who reported BDH indicated that no corrective action was taken.

Prior surveys have highlighted the troubling prevalence of BDH within the medical field.^3^, ^4^, ^5^ In cardiology, these issues can be compounded by the high-stress nature of the work, organizational hierarchy, and historically entrenched biases mainly against women, in a largely male-dominated profession.^6^^,^^7^ According to a survey conducted by the American College of Cardiology (ACC), a significant proportion of cardiologists, particularly women report experiencing discrimination or harassment at some point in their careers.^8^ Another global survey by ACC indicated that of the 5931 cardiologists including 23% women, 44% reported a hostile work environment.^3^ In that survey, discrimination (56% women vs 22% men), emotional (43% women vs 26% men), and sexual (12% women vs 1% men) harassment were significantly more common in women. In the UK survey by Jaijee et al^5^, 61.9% female cardiologists reported any kind of discrimination over their professional careers. In the U.S. survey by Lewis et al^8^, 65% women cardiologists reported experiencing career discrimination in 2015. In the Italian survey of interventional cardiologists by Bernelli et al^9^, 62.8% women reported some type of workplace discrimination, and 90.7% women felt female gender was discriminatory for career purposes. Compared to these surveys, our snapshot survey suggests a higher rate of discrimination (90%) at any time over the careers of respondents. Our survey noted that 31.4% women reported sexual harassment at any time over their careers. Prior surveys have also shown that one-third of women cardiologists have experienced sexual harassment.^5^^,^^8^ In addition, in our survey, compared to senior CSANZ fellows of >10 years duration, junior CSANZ fellows of <10 years duration reported significantly greater prevalence of comments about culture or race that made them feel uncomfortable.

In Australia, the regulatory bodies, Medical Board of Australia and AHPRA have conducted medical training surveys of doctors in training across different specialties since 2019.^10^ Staff specialists or consultants are not included in the survey. In the 2024 survey, 36% trainees across all specialties reported experiencing or witnessing BDH or racism within 12 months. Similar to our findings, in the RACP college-specific report, 20% trainees reported that they did not know how to raise concerns about BDH. However, our exploratory analyses showed that there was no association between awareness of BDH or having someone to talk to, and reporting to a relevant authority. In additon, one-third of those trainees who experienced or witnessed BDH were not satisfied that their report was followed up, a higher proportion compared to the results in our survey (17.1%).

The current pilot survey was limited to distribution via the WIC mailing list and WIC dinner meeting, representing only a small sample of women in cardiology across Australasia. The overall number of WIC members (across all subspecialties and including consultants, trainees, nursing, and allied health staff) in the region has not been systematically documented. The response rate was moderate, but better than other such professional surveys (ACC global survey 8.35%; United Kingdom, 19.6%; United States, 21%; Italy, 26.5%).^3^^,^^5^^,^^8^^,^^9^

The free-text responses in our study highlighted entrenched power imbalances, bullying, and systemic discrimination that create an environment where individuals feel powerless and excluded from decision-making. Toxic leadership, factional infighting, and a fear-driven culture prevent trainees and colleagues from lodging complaints, as doing so could jeopardize their careers. Gender discrimination is deeply embedded in training structures and personal lives are scrutinized. Many believe governing bodies such as CSANZ and RACP have been ineffective. The reluctance of colleagues to call out misconduct protects those in power, perpetuating the cycle of bullying, mistreatment, and trauma. One-third of medical trainees in the medical training RACP college-specific survey showed concerns about repercussions of reporting, 18% were concerned by the lack of processes, and 14% did not know who to report to.^10^

The data from the current study indicate a need for deeper investigation and robust action plans to address the problem. This study also provides some insights into the type of BDH behaviors that are likely to be associated with higher rates of reporting to a relevant authority suggesting a gradient of less severe to more severely damaging experiences among victims. A structural change is essential as victims of these behaviors can suffer severe psychological effects, including anxiety, depression, burnout, and absent job satisfaction, resulting in elimination from the workforce. In contrast, ongoing work as usual in the context of unaddressed negative experiences can affect clinical decision-making, patient care, and safety. Developing effective strategies to prevent and mitigate BDH requires all stakeholders to work together.

By way of developing solutions, the ACC writing committee in 2022 published a health policy statement on building respect, civility, and inclusion in the workplace.^11^ In Australasia, a similar approach is crucial with a view to creating a code of ethics and holding members accountable. However, enforcement of these guidelines can be challenging in the absence of a clear and uniform legal and regulatory framework applicable across all institutions.

Measures to prevent and manage BDH: The Central Illustration demonstrates a summary of the problem and the proposed measures to change the status quo.

a. Male allyship initiatives: Cardiology continues to be a male-dominated specialty, and between 2015 and 2017, women represented only 15% of the specialist cardiology workforce in Australia and New Zealand.^12^ To address this imbalance, male allyship programs, aligned with global movements like the United Nations HeForShe solidarity campaign, can play a pivotal role in fostering equity and inclusion within the profession.^13^
b. Reporting and resolutions: A clear pathway for anonymous reporting remains an unaddressed issue. We propose the establishment of a systematic reporting process facilitated through elected anti-BDH representatives within each institution. These representatives should be empowered to liaise with hospital administration and collaborate across institutions to develop resolutions and seek support while protecting the victims. Hospitals, AHPRA, RACP, and CSANZ must develop and implement safer, lower-risk complaint processes to protect those who report misconduct, where inappropriate behavior is addressed early through brief but firm interventions by leadership before escalation. A dedicated Wellbeing Officer within CSANZ could provide members with support and an independent avenue to anonymously report misconduct. In Australia, sexual harassment cases can be reported to the workplace, the Fair Work Ombudsman, the Fair Work Commission or the Australian Human Rights Commission.^14^ However, a single national authority to oversee all cases is desirable, as described by Haskell et al.^1^ An annual summary report on BDH reports and reviews, as detailed by Douglas et al^11^ could then be communicated to members.
c. Creating culture change: Organizational culture and climate may be one of the greatest indicators of the occurrence of BDH.^15^ Mansour et al^16^ highlight the importance of understanding implicit societal biases and victim blaming in cases of sexual harassment, which may prevent victims from reporting sexual harassment but also other forms of BDH. Implicit bias training and bystander training can change the victim-blaming culture and allow more individuals to stop and report witnessed harassment.^17^ In Australia, the Fair Work Commission provides a free online training course to help individuals respond to witnessed or experienced workplace sexual harassment.^18^ Moreover, knowledge and discussion of BDH research will allow women to interrupt, discourage, and report BDH as it happens.^14^^,^^19^ The ACTION framework by Bravo-Jaimes et al^20^ adapted from Douglas et al^11^ (Ask clarifying questions, be Curious, Tell the observed facts, Intention vs impact, Own your thoughts, Next steps) provides a practical guide to assist with early conversations between targets and perpetrators to curb BDH before the need for further escalation.
d. Accreditation: Accreditation of cardiology training programs by RACP is a powerful tool and should be leveraged as a motivator for hospital reform. Reports of BDH within an institution should be recorded, and failure to take corrective action should result in penalties, particularly at the time of reaccreditation for new trainees.
e. Membership and registration: Members of the RACP and CSANZ should be made aware of BDH and strict disciplinary actions should be outlined to tackle these behaviors through annual policy training. All leadership must emphasize a zero-tolerance policy for BDH. Disciplinary action should include suspension or cancellation of membership. Reporting BDH to AHPRA via mandatory notification or a confidential complaint pathway will allow review of cases, and appropriate regulatory action or referral for further investigation.^21^
f. Mentorship: Mentorship programs fostered within and outside local institutions can provide a platform to safely discuss BDH and help victims seek support in parallel to the formal resolution process, notably outside the parent organization.
g. Gender neutral policies: RACP, CSANZ, and hospitals must provide guidance on the inappropriateness of gender-biased questions at recruitment interviews. In addition, equal job-sharing policies for men and women are essential. Breaking gender stereotypes with a gender-neutral policy offering paid leave and flexible working conditions must be an explicit requirement for accreditation of institutions.
h. Ongoing surveys and research: Regular surveys can track progress and accountability. These survey reports should become a key performance indicator for training programs, ensuring institutions foster a safe and supportive environment. Cultural change can only take place with open dialogue at all levels. The competitive, hierarchical nature of cardiology must be re-examined so that fear no longer dictates whether individuals can seek support. More research on BDH is required to understand the complex nature of the problem and to test the effectiveness of proposed solutions.^11^

**Central Illustration:**
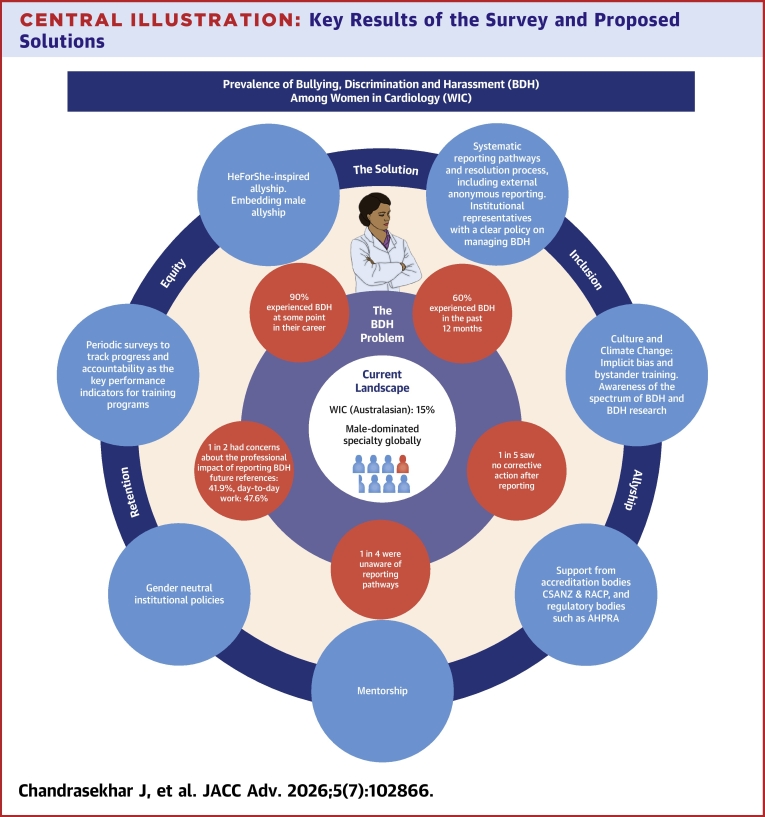
Key Results of the Survey and Proposed Solutions AHPRA = Australian Health Practitioner Regulation Agency; CSANZ = Cardiac Society of Australia and New Zealand; RACP = Royal Australasian College of Physicians.

### Study Limitations

Our study has limitations. The survey was only sent to a sample of women in cardiology and included participants from 2 events, which may lead to selection bias and may not be representative of the larger WIC group across Australasia. However, the findings are aligned with other similar surveys. The survey sample under-represents trainees and allied health professionals and as such, results may not be generalizable to these groups. Our survey did not capture the overlapping dynamics of intersectionality. We did not capture nonrespondents and acknowledge that nonresponse bias is a limitation of this survey; therefore, findings should be considered exploratory. Although BDH is more commonly reported by women, men also experience these biases in the workplace, and their experiences should be documented and reported. Following this pilot survey, we anticipate a larger interval survey inviting all specialist consultants and trainees in cardiology to participate and report on the problem.

## Conclusions

BDH was noted to be highly prevalent from this snapshot survey of women in cardiology with 90% reporting the experience of these behaviors anytime during their career. One in 4 were unaware of how to report this but nearly 1 in 5 who reported the issue saw no consequent corrective actions. Urgent measures are required to reduce and eradicate these negative behaviors from cardiology training and workplaces, a process that requires significant input and change from all stakeholders.

## Funding support and author disclosures

The authors have reported that they have no relationships relevant to the contents of this paper to disclose.
