# Supplementary material for: Prevalence of Bullying, Discrimination, and Harassment Among Women in Cardiology: A Snapshot Australasian Survey
Source: JACC Adv. 2026 Jul 22;5(7):102866. doi: 10.1016/j.jacadv.2026.102866 (PMC13400111; doi:10.1016/j.jacadv.2026.102866)
Supplement: Supplemental Material [file mmc1.docx]

**Supplementary Table 1: Prevalence estimates for Bullying, Discrimination, and Harassment behaviours ever experienced by respondents during their cardiology careers**

| **Thinking about your workplace, have you personally experienced or witnessed in your department any of the following behaviours ever in your cardiology career? (Please tick all that apply)** | **Mean** | **95% Confidence Interval lower, upper limit** | **Standard error** |
| --- | --- | --- | --- |
| Aggression or physical abuse | 21% | 13, 29% | 0.040 |
| Being undermined / humiliating comments made about me or towards me or a colleague when alone | 60% | 50, 70% | 0.048 |
| Humiliating comments made about me or towards me or a colleague in front of others | 53% | 44, 63% | 0.049 |
| Inappropriate criticisms/accusations/ belittling behavior | 63% | 53, 72% | 0.047 |
| Yelling or shouting | 42% | 32, 51% | 0.048 |
| Unwelcome advances of a sexual nature (breach of personal space) | 31% | 22, 40% | 0.046 |
| Being denied operating lists or procedures | 12% | 6, 19% | 0.032 |
| Being denied training opportunities | 24% | 16, 32% | 0.042 |
| Being excluded from meetings related to my role | 16% | 9, 23% | 0.036 |
| Being assigned meaningless tasks | 19% | 11, 27% | 0.039 |
| Being excluded from social events where other colleagues have been invited | 30% | 21, 38% | 0.045 |
| Being denied a promotion | 15% | 8, 22% | 0.035 |
| Being denied an interview opportunity for a leadership role | 9% | 3, 14% | 0.027 |
| Receiving favorable/unfavorable treatment because of gender/race | 29% | 20, 37% | 0.044 |
| Comments about my culture or race that made me feel uncomfortable | 14% | 7, 21% | 0.034 |
| Other unwelcome behavior | 17% | 10, 24% | 0.037 |
| Opinions and views ignored | 39% | 30, 49% | 0.048 |
| Pressured into not claiming something to which you were entitled (eg overtime payment, leave, reimbursement, increased paygrade, faculty position) | 48% | 38, 57% | 0.049 |
| Suggested to quit job/ training program | 9% | 3, 14% | 0.027 |
| None of the above | 10% | 5, 16% | 0.030 |

**Supplementary Table 2: Prevalence estimates for Bullying, Discrimination, and Harassment behaviours experienced in the previous 12 months by respondents**

| **Thinking about your workplace, have you personally experienced or witnessed in your department any of the following behaviours in the last 12 months? (Please tick all that apply)** | **Mean** | **95% Confidence Interval lower, upper limit** | **Standard error** |
| --- | --- | --- | --- |
| Aggression or physical abuse | 4% | 0, 8% | 0.019 |
| Being undermined / humiliating comments made about me or towards me or a colleague when alone | 30% | 21, 38% | 0.045 |
| Humiliating comments made about me or towards me or a colleague in front of others | 25% | 16, 33% | 0.042 |
| Inappropriate criticisms/accusations/ belittling behavior | 25% | 16, 33% | 0.042 |
| Yelling or shouting | 13% | 7, 20% | 0.033 |
| Unwelcome advances of a sexual nature (breach of personal space) | 6% | 1,10% | 0.023 |
| Being denied operating lists or procedures | 8% | 2, 13% | 0.026 |
| Being denied training opportunities | 8% | 2, 13% | 0.026 |
| Being excluded from meetings related to my role | 9% | 3, 14% | 0.027 |
| Being assigned meaningless tasks | 11% | 5, 18% | 0.031 |
| Being excluded from social events where other colleagues have been invited | 7% | 2, 12% | 0.024 |
| Being denied a promotion | 9% | 3, 14% | 0.027 |
| Being denied an interview opportunity for a leadership role | 6% | 1,10% | 0.023 |
| Receiving favorable/unfavorable treatment because of gender/race | 12% | 6, 19% | 0.032 |
| Comments about my culture or race that made me feel uncomfortable | 6% | 1,10% | 0.023 |
| Other unwelcome behavior | 10% | 5, 16% | 0.030 |
| Opinions and views ignored | 19% | 11, 27% | 0.039 |
| Suggested to quit job/ training program | 2% | -1, 5% | 0.013 |
| Pressured into not claiming something to which you were entitled (eg overtime payment, leave, reimbursement, increased paygrade, faculty position) | 14% | 7,21% | 0.034 |
| None of the above | 41% | 31, 51% | 0.048 |

**Supplementary Table 3: Differences in Bullying, Discrimination, and Harassment behaviours ever experienced between CSANZ fellows of <10 or >10 years duration**

| **Thinking about your workplace, have you personally experienced or witnessed in your department any of the following behaviours ever in your cardiology career? (Please tick all that apply)** | **Fellow for <10 years**  **(n=48)** | **Fellow for > 10 years**  **(n=34)** | **p-value** |
| --- | --- | --- | --- |
| Aggression or physical abuse | 16.67% | 26.47% | 0.281 |
| Being undermined / humiliating comments made about me or towards me or a colleague when alone | 68.75% | 52.94% | 0.146 |
| Humiliating comments made about me or towards me or a colleague in front of others | 56.25% | 47.05% | 0.412 |
| Inappropriate criticisms/accusations/ belittling behavior | 58.33% | 67.65% | 0.392 |
| Yelling or shouting | 37.50% | 35.29% | 0.838 |
| Unwelcome advances of a sexual nature (breach of personal space) | 29.17% | 32.35% | 0.757 |
| Being denied operating lists or procedures | 12.50% | 14.71% | 0.773 |
| Being denied training opportunities | 29.17% | 17.65% | 0.231 |
| Being excluded from meetings related to my role | 14.58% | 17.65% | 0.708 |
| Being assigned meaningless tasks | 17.65% | 20.83% | 0.720 |
| Being excluded from social events where other colleagues have been invited | 29.17% | 29.41% | 0.981 |
| Being denied a promotion | 6.25% | 20.59% | 0.084 |
| Being denied an interview opportunity for a leadership role | 10.42% | 5.88% | 0.694 |
| Receiving favorable/unfavorable treatment because of gender/race | 31.25% | 26.47% | 0.639 |
| Comments about my culture or race that made me feel uncomfortable | 20.83% | 2.94% | *0.022 |
| Other unwelcome behavior | 16.67% | 8.82% | 0.348 |
| Opinions and views ignored | 35.42% | 47.06% | 0.290 |
| Pressured into not claiming something to which you were entitled (eg overtime payment, leave, reimbursement, increased paygrade, faculty position) | 56.25% | 44.11% | 0.279 |
| Suggested to quit job/ training program | 8.33% | 5.88% | 1.000 |
| None of the above | 8.33% | 11.76% | 0.713 |
